# Warming Accelerates Phytoplankton Bloom Dynamics and Differentially Affects the Fluxes of Carbon, Nitrogen, and Oxygen Through a Coastal Microbial Community

**DOI:** 10.1007/s00248-025-02643-9

**Published:** 2025-11-07

**Authors:** Daffne C. López-Sandoval, Cristina Fernández-González, Cristina González-García, Emilio Marañón

**Affiliations:** 1 Coastal and Marine Resources Core Lab (CMR), King Abdullah University of Science and Technology (KAUST), 23955 Thuwal, Saudi Arabia; 2 Centro de Investigación Mariña, Universidade de Vigo (CIM-UVigo), 36331 Vigo, Spain; 3 Department of Ecology and Animal Biology, University of Vigo, 36310 Vigo, Spain

**Keywords:** Carbon-to-nitrogen uptake ratio, Microbially driven ecosystem, Coastal microbial community, Marine heatwaves (MHWs), Short-term warming events, Nutrient availability

## Abstract

Marine heatwaves affect the abundance and community structure of microbial plankton, with implications for food web and ecosystem processes, but their impact on microbially mediated elemental cycling remains poorly constrained. To determine the biogeochemical effects of increased temperature, we conducted an experiment in September 2023 in which a plankton community from a coastal, productive ecosystem (Ría de Vigo, NW Iberia) was exposed to a warming of + 2 °C and + 4 °C under unamended and nutrient-enriched conditions. The response of microbial plankton was characterized in terms of organic matter production, carbon fixation, nitrogen uptake, and oxygen net production. We found that warming caused increased nutrient consumption and biomass production, as well as faster bloom dynamics, both in unamended and nutrient-enriched treatments, indicating that the community was robust to thermal perturbation. Accelerated nutrient depletion under warming gave way to an earlier decrease in carbon fixation and nitrate uptake rates, together with a shift towards a negative or less positive metabolic balance. Carbon fixation was less sensitive than nitrate uptake to the different temperature and nutrient scenarios, leading to wide changes in the carbon-to-nitrogen uptake ratio, while respiration increased non-linearly with temperature. Overall, the investigated microbial fluxes were more responsive to nutrient availability than to temperature. Our results show that microbially driven ecosystem services in coastal waters have the potential to be enhanced during short-term warming events.

**Supplementary Information:**

The online version contains supplementary material available at 10.1007/s00248-025-02643-9.

## Introduction

Marine heatwaves (MHWs), defined as events during which seawater temperature exceeds the climatological 90th percentile value for at least five consecutive days [1], are growing in frequency, duration, and intensity [2–4]. This trend has accelerated recently, as the annual number of MHW days in the ocean has doubled between 1982 and 2016 and is expected to increase further by a factor of 16–23 with a global warming of 1.5–2.0 °C above preindustrial temperature [4]. The biological effects and resulting socio-economic impacts of MHWs have been well documented for coral reefs, kelp forests, seagrass meadows, mangroves, invertebrates, and fish [5–8]. Comparatively less is known about the impacts on microorganisms, even though they control marine biogeochemical cycles and the provision of numerous ecosystem services [9]. For instance, the annual net primary production by marine phytoplankton (48–52 GtC year^−1^) [10] nearly equals that of land plants [11] and drives the biological carbon pump, whereby CO_2_ fixed into organic matter is exported to the ocean’s interior at a rate of 5–12 GtC year^−1^ [12]. Microbial plankton communities also play a key role in alleviating coastal water pollution [13] and supporting the transfer of food and energy towards upper trophic levels including fisheries [14, 15]. Hence, investigating the effect of climate variables on microbial plankton communities and their associated elemental cycling is a major research priority in global change biology [16, 17].

The effects of MHWs on phytoplankton biomass and bloom dynamics, based on chlorophyll *a* estimates from remote sensing of ocean colour or in situ autonomous instruments, have been investigated at global [18, 19] and regional [20–22] scales. An emerging pattern from these studies is that heatwaves cause an increase in phytoplankton biomass when nutrient availability is high, and a decrease when nutrients are scarce [18, 19, 21, 22]. In addition, when MHWs lead to decreased phytoplankton biomass, a shift towards increased dominance by smaller species is typically observed, both with remote sensing [20, 23] and in situ [24–26] approaches, which reflects the competitive advantage provided by small cell size under conditions of low nutrient availability [27, 28]. Observations of prokaryotic diversity during MHWs have also revealed profound community reorganizations in both archaea and bacteria, with increased presence of taxa associated with warm and nutrient-poor conditions [29, 30]. However, given the inverse correlation between temperature and nutrient concentration in the upper ocean [31], observational studies must be complemented with experimental approaches in order to disentangle and quantify the independent and interactive biological effects of these drivers [17, 32].

Temperature-nutrient manipulative experiments have shown that nutrient availability modifies the response to warming and, in turn, changes in temperature affect the response to nutrient supply across multiple levels of biological organization in both coastal [33–35] and open-ocean [36, 37] microbial plankton communities, as well as in laboratory populations [38, 39]. A common result from these studies is that enhanced nutrient availability leads to increased warming-induced phytoplankton growth, while nutrient limitation causes reduced temperature sensitivity of metabolic rates [34, 35, 38, 39]. The relatively low thermal sensitivity of phytoplankton growth and productivity in oligotrophic environments has also been confirmed in field studies [40–42]. Conversely, warming modulates the response to nutrient availability at the cellular level, synergistically increasing light-harvesting capacity [37, 38], and at the community level, leading to increased trophic transfer under low-nutrient conditions [33]. In spite of these advances, there is still little understanding of how the interaction and feedback between temperature and nutrient availability regulate the microbial cycling of elements [17]. Considering simultaneously multiple elements such as carbon, nitrogen, and oxygen is needed to assess the biogeochemical impacts of warming episodes through changes in primary productivity, metabolic balance, and the stoichiometry of newly produced organic matter, all of which can affect critical ecosystem services including nutrient removal, transfer of organic matter towards upper trophic levels, and CO_2_ sequestration.

To investigate the effects on photosynthetic carbon fixation, nitrogen uptake, and the metabolic balance between photosynthesis and respiration, we conducted an experiment in September 2023 in which a natural microbial plankton community from Ría de Vigo (NW Iberian Peninsula) was exposed to three different temperatures (in situ and warming by 2 °C and 4 °C) under two nutrient regimes (unamended and addition of N, P, and Si). Ría de Vigo is the southernmost ría of the Rías Baixas, highly dynamic and productive coastal embayments located in the northern limit of the upwelling system off Iberia-NW Africa that support intense fishing and mariculture activities [43–45]. We anticipate that warming will lead to increased phytoplankton growth and metabolic rates, and that this stimulation will be stronger under nutrient-enriched conditions. However, because of the different thermal sensitivities of various metabolic and trophic processes, additional specific hypotheses can be put forward. The dark reactions of photosynthesis have a stronger temperature dependence than that of diffusion-limited nutrient transport across the cell membrane [46, 47], and therefore CO_2_ fixation can be expected to increase with temperature more rapidly than nutrient uptake. Depending on whether phytoplankton stoichiometry is primarily controlled by temperature or by nutrient availability [48–50], the carbon-to-nitrogen ratio of the newly produced organic matter may differ among the various temperature-nutrient scenarios. Respiration has a stronger temperature dependence than photosynthesis [51, 52], which means that (if the supply of organic substrates does not become limiting for heterotrophs) the net metabolic balance of the community should decrease under warming conditions. Similarly, if the growth of protist grazers is more sensitive to temperature than that of photoautotrophs [53], warming could lead to stronger top-down control of phytoplankton, resulting in a weaker bloom. By examining these hypotheses, we aim to advance our understanding of the role of temperature and nutrients in the control of photosynthetic carbon fixation, nutrient uptake, and net community production in the surface ocean, and to assess the sensitivity to short-term warming events of the base of the food web in a marine ecosystem with high socioeconomic value.

## Methods

### Sampling and Experimental Setup

We visited a station in the main channel of Ría de Vigo (NW Iberian Peninsula; 42° 14.1′ N, 8° 46.8′ W; depth = 45 m) aboard R/V *Mytilus* on 11 September 2023. Prior to sampling, the vertical distribution of temperature, salinity, and chlorophyll *a* fluorescence was determined with a SeaBird 25CTD probe attached to a rosette. Using 12-L Niskin bottles, 80 L of surface (1–2 m depth) seawater were collected and then transferred to acid-washed polypropylene 20-L carboys. Once in the laboratory, unfiltered seawater was dispensed into eighteen acid-washed 4-L Nalgene polycarbonate bottles, which were placed in an outdoor recirculating-water incubator equipped with three 90-L tanks whose temperature was controlled individually. The tanks were covered with a neutral density mesh that removed 40% of incident natural irradiance. The experimental treatments consisted of all combinations of three temperatures (in situ 18.6 °C, 20.6 °C, and 22.6 °C) and two nutrient conditions (unamended and addition of N, P, and Si), all carried out in three biological replicates (3 × 2 × 3 = 18 experimental units). The nutrient-amended bottles were spiked with NH_4_NO_3_, Na_2_SiO_3_, and Na_2_HPO_4_ to give a nominal concentration increase of 6 µmol L^−1^ NO_3_^−^, 6 µmol L^−1^ NH_4_^+^, 0.2 µmol L^−1^ HPO_4_^2−^, and 4 µmol L^−1^ H_4_SiO_2_. The resulting nutrient concentrations are within the range of values reported for subsurface waters of Ría de Vigo in late summer [43, 54], which could be brought towards the surface during an upwelling event. The concentration of different standing stocks and microbially mediated fluxes of carbon, nitrogen, and oxygen were measured at the start and at the end of the experiment, which lasted for 5 days (including the 24-h incubation period to determine metabolic rates on the last day). For some variables (see the “Results” section), measurements were also conducted daily or every 2 days. The 5-day duration of the experiment is commensurate with the typical flushing time of Ría de Vigo (3–5 days during spring–summer) [55, 56].

### Standing Stocks

The concentration of inorganic nutrients (nitrate, nitrite, phosphate, and silicate) and chlorophyll *a* (chl *a*) was determined at the start (Initial conditions) and at the end of the experiment and also after 2 days of incubation. After collection in 20-mL polypropylene tubes, nutrient samples were frozen and stored at − 80 °C until spectrophotometric analysis on a Skalar segmented-flow analyser. The limits of detection were 0.02 µmol L^−1^ for nitrate, phosphate, and silicate, and 0.01 µmol L^−1^ for nitrite. For the determination of chl *a*, 250-mL samples were filtered onto Whatman GF/F filters, which were stored at − 80 °C until analysis. Pigment extraction was carried out by placing the filters in 90% acetone for 12 h, after which fluorescence was measured on a TD-700 Turner fluorometer that had been calibrated with pure chl *a*. The concentration of particulate organic carbon (POC) and nitrogen (PON) was measured as part of the procedure to determine the rates of CO_2_ fixation and NO_3_ uptake using the isotopes ^13^C and ^15^N (see below).

The abundance of picoplankton (heterotrophic bacteria, *Synechococcus*, and small and large picoeukaryotes) was determined with a Beckman Coulter Cytoflex flow cytometer equipped with 488-nm and 633-nm excitation wavelength lasers. Samples (1.75 mL) were fixed with 1% paraformaldehyde + 0.05% glutaraldehyde (final concentration), incubated for 10 min in the dark, flash-frozen with liquid N_2_, and stored at − 80 °C until analysis. Prior to analysis, samples were thawed at 5 °C for 4–8 h and then left at room temperature for 30 min. The cytometer flow rate was set at 60 µL min^−1^ and the sampling time was 2 min. The abundance of the different populations was determined from their autofluorescence (> 650 nm and 585 nm emission for chl *a* and phycoerythrin, respectively) and forward scattering signals. Plots of forward scattering versus phycoerythrin fluorescence were used to enumerate *Synechococcus*, and plots of scattering versus chl *a* fluorescence were used to count picoeukaryotic algae. The forward scattering signal was calibrated using six different sizes of beads (1–15 µm in diameter). For the analysis of heterotrophic bacteria, samples were stained with 15 µL of SybrGreen DNA fluorochrome. The cytometer flow rate was set at 10 µL min^−1^ and the sampling time was 2 min. Total bacterial abundance was determined from the green fluorescence (519 nm emission) and forward scatter signals. The biomass of *Synechococcus* and picoeukaryotes was estimated from their abundance and biovolume by applying the volume to biomass conversion factors of [57]: 230 fgC µm^−3^ for *Synechococcus* and 237 fgC µm^−3^ for picoeukaryotes. To calculate the biomass of heterotrophic bacteria, we used a cell-specific carbon content of 20 fgC cell^−1^ [58].

### Metabolic Rates

We conducted dual-labelling experiments with ^13^C and ^15^N to determine the rates of photosynthetic carbon fixation and nitrate uptake from the heavy-isotope enrichment of organic matter during the incubation [59, 60]. Samples (0.5 L in volume) were collected in acid-washed Nalgene polycarbonate bottles and spiked with heavy isotope-enriched (98–99%) solutions of NaH^13^CO_3_ and Na^15^NO_3_ at final concentrations of 92 µmolC L^−1^ and 0.01–0.02 µmolN L^−1^, respectively. Bottles were incubated for 24 h in the same temperature-controlled tanks described above. An additional 0.5-L sample was collected from each experimental treatment and processed immediately to determine ^15^N and ^13^C natural abundance and initial POC and PON concentration. We also incubated one isotope-enriched bottle from each treatment under dark conditions. Assuming a constant concentration of dissolved inorganic carbon (DIC) of 2140 µmol L^−1^, as reported for the sampling station [61], the ^13^C addition resulted in a DIC increase of 4.3% over ambient levels. In the case of nitrate, the ^15^N addition caused an increase of 55% over ambient (initial) levels in the unamended treatments at 20.6 °C and 22.6 °C, and < 16% in all the other treatments. Incubations were terminated by filtration, using low vacuum pressure (< 100 mmHg), through pre-combusted Whatman GF/F filters that were then stored at − 80 °C until analysis. After thawing, filters were exposed to concentrated HCl fumes overnight, maintained in a desiccator for 24 h, and then packaged into tin foil capsules. The measurement of POC, PON, δ^13^C, and δ^15^N in the filters was conducted by combustion on a FlashEA1112 (ThermoFinnigan) elemental analyser coupled through a ConfloIII interface to a MAT253 (ThermoFinnigan) isotope-ratio mass spectrometer. The rates of photosynthetic carbon fixation and NO_3_^−^ uptake were computed with the formulas described in [62]. Carbon fixation rates in light and dark bottles were calculated from the difference in ^13^C atom % between the final and the initial bottles. To obtain the rate of photosynthetic carbon fixation, the rate measured in the dark bottles was subtracted from the rate measured in the light bottles. NO_3_^−^ uptake rate was calculated from the difference in ^15^N atom % between the incubated (light bottles) and the initial samples. Specific carbon fixation and nitrate uptake rates were calculated by dividing the volumetric rates by chl *a* concentration.

Net community production (NCP) and community respiration (CR) were determined with the light and dark bottle O_2_ evolution method. We used 40-mL borosilicate bottles provided with Presens SP-PSt3 sensor spots, and O_2_ concentration measurements were conducted with an optical fiber connected to a Presens Fibox 4 trace instrument. Sensors were calibrated with a two-point calibration, where oxygen-free water was obtained with a solution of Na_2_SO_3_ and Co(NO_3_)_2_, while water with 100% O_2_ saturation was obtained by bubbling air with an air pump. Measurements were conducted with the automatic salinity compensation provided by the Fibox 4 software. For each microcosm (biological replicate), three bottles (technical replicates) were filled with seawater to determine initial O_2_ concentration, [O_2_]_i_, two bottles were incubated in the light, and two bottles (covered with black tape) were incubated in the dark. After 24 h of incubation, the final O_2_ concentration, [O_2_]_f_, was measured (5–6 readings per sample over 30–40 s) in all bottles. NCP was computed as [O_2_]_f_ in the light bottles minus [O_2_]_i_, while CR was determined as [O_2_]_i_ minus [O_2_]_f_ in the dark bottles. Gross primary production (GPP) was calculated as the sum of NCP and CR. Specific NCP, CR, and GPP rates were calculated by dividing the volumetric rates by the POC concentration.

We assessed the photochemical quantum efficiency of photosystem II using pulse-amplitude modulated (PAM) fluorometry. Duplicate 5-mL samples were collected from each microcosm, transferred to Falcon tubes, and maintained in the dark for 30 min, after which initial fluorescence (*F*_o_) and maximal fluorescence (*F*_m_) were measured with a Phyto-PAM II fluorometer (Walz). The maximum potential quantum efficiency of photosystem II was calculated as *F*_v_/*F*_m_ = (*F*_m_ − *F*_o_)/*F*_m_.

### Data Analysis and Statistics

We used the Kruskal–Wallis test and a posteriori Dunn’s test to assess the differences among temperature treatments for each nutrient treatment and sampling day. The combined effects of temperature, nutrient regime, and their interaction on standing stocks and metabolic rates at the end of the experiment were assessed with the Aligned Rank Transform test (ARTool version 0.11.1). All statistical analyses were carried out with R Studio (Version 2024.12.1 + 563).

## Results

### Initial Conditions

During 1 week prior to sampling, negative values of the upwelling index indicated downwelling-favourable conditions, and thus shoreward transport of surface water (Fig. S1). Accordingly, on the sampling day, sea surface temperature was warm (18.6 °C), thermal stratification was strong, and surface chl *a* was low (2.5 µg L^−1^) (Fig. S2). The surface concentrations of nitrate, nitrite, phosphate, and silicate were 0.51, 0.15, 0.29, and 1.17 µmol L^−1^, respectively (Table S1).

### Nutrients and Chlorophyll *a*

**Fig. 1:**
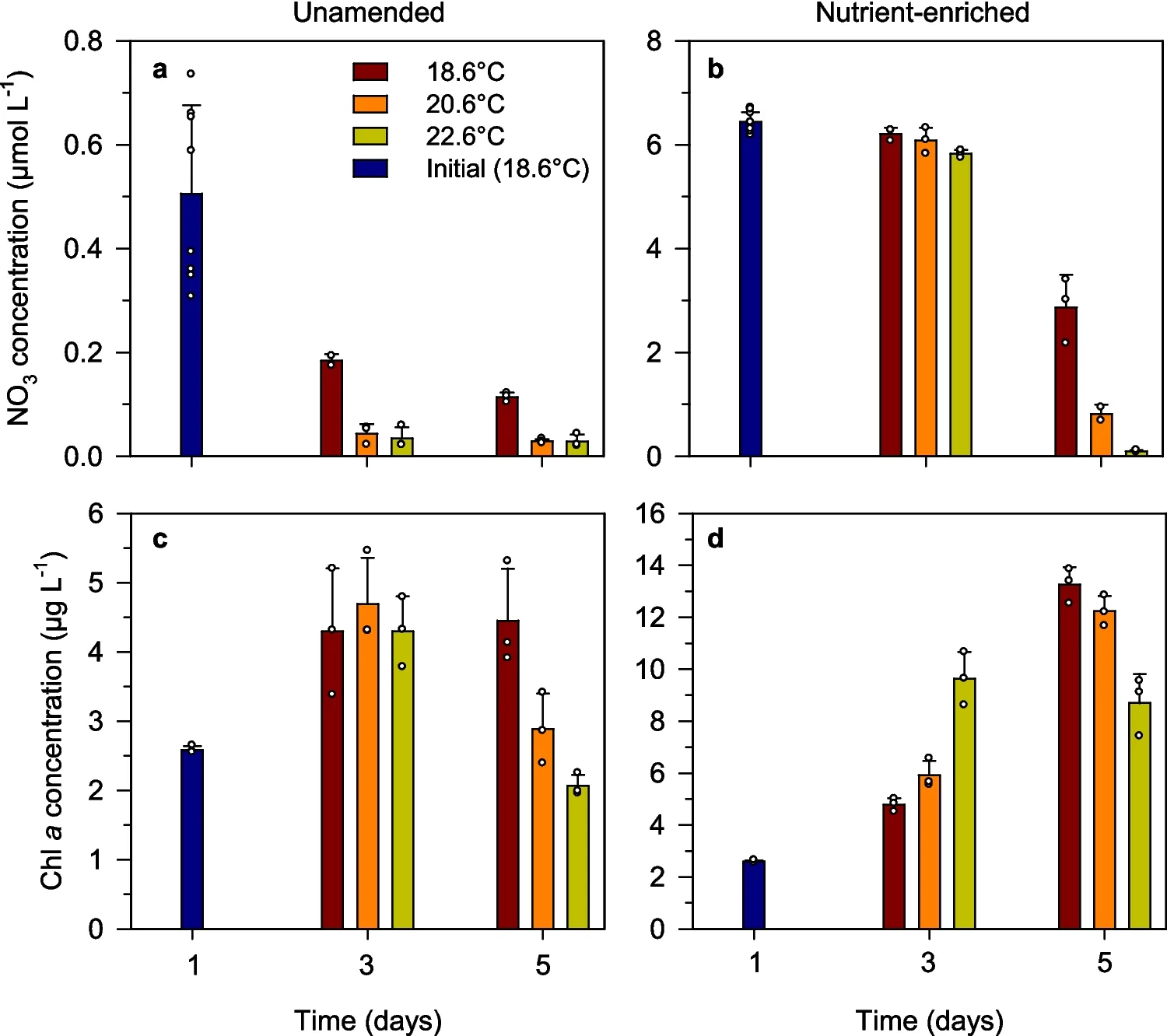
Temporal variability of **a**, **b** nitrate concentration and **c**, **d** chlorophyll *a* concentration in the different temperature treatments in **a**, **c** unamended and **b**, **d** nutrient-enriched microcosms. Note the differences in the y-axis scale between the left panels (unamended treatments) and the right panels (treatments with added nutrients). Values shown are the mean of three biological replicates, and error bars are the standard deviation. Small dots represent the values of each biological replicate

We observed a consistent pattern of nitrate consumption and chl *a* increase (in some cases, followed by a decrease) over time in all experimental units, but the magnitude and timing of these changes differed markedly between treatments (Fig. 1, Table S1). Compared with the control (in situ) temperature, nitrate concentration in unamended bottles was lower in the warmed treatments, both on days 3 and 5 (Fig. 1a). In the nutrient-enriched treatments, the effect of temperature on nitrate consumption was still minor on day 3 but became apparent by day 5, when nitrate concentration was progressively lower with increasing temperature (Fig. 1b). From day 3 to day 5, net nitrate consumption was approximately 3, 5, and 6 µmol L^−1^ at 18.6 °C, 20.6 °C, and 22.6 °C, respectively. Nitrite concentrations were always ≤ 0.15 µmol L^−1^ and showed comparatively minor differences between temperature treatments (Table S1). The concentration of phosphate ranged between 0.2 and 0.5 µmol L^−1^ during days 1 and 3, but increased on the last day of the experiment, particularly at 18.6 °C and 20.6 °C, reaching values ≥ 0.8 µmol L^−1^. Silicate concentration in the enriched treatments remained relatively unchanged from day 1 to day 3 (4.5–5.5 µmol L^−1^), then decreased to values ≤ 1.3 µmol L^−1^ on day 5, with this decrease being larger under warmer temperature (Table S1). This significant silicate net consumption rate (approximately 2 µmol L^−1^ day^−1^) suggests that diatoms contributed to the increased phytoplankton biomass in response to nutrient addition.

As expected, the largest chl *a* accumulation took place in nutrient-amended treatments, where final concentrations of 12–13 µg L^−1^ were measured at 20.6 °C and 22.6 °C (Fig. 1d), representing a fivefold increase from the initial value of 2.5 µg L^−1^. However, substantial (ca. 80%) increases in chl *a* also occurred in unenriched treatments at all temperatures (Fig. 1c). Temperature affected the pace of chl *a* accumulation and subsequent decline, but this effect was in turn dependent on the nutrient regime. On day 3, the different temperature treatments in unamended microcosms had similar chl *a* (Fig. 1c), whereas under nutrient-enriched conditions, chl *a* was progressively larger with increasing temperature, with the fastest chl *a* increase (by a factor of > 3) occurring at the warmest temperature. We found strong evidence for this interactive temperature (*T*) × nutrient (*N*) effect on chl *a* accumulation (ART test, *F*_*T* × *N*_ = 32.8, *df* = 2, *p* < 0.0001). Similarly, chl *a* concentration on day 5 was progressively smaller with increasing temperature in both nutrient regimes (ART test, *F*_T_ = 40.7, *df* = 2, *p* < 0.0001), suggesting that the warmer the temperature, the more advanced the bloom development and subsequent decline.

### Particulate Organic Carbon and Nitrogen

**Fig. 2:**
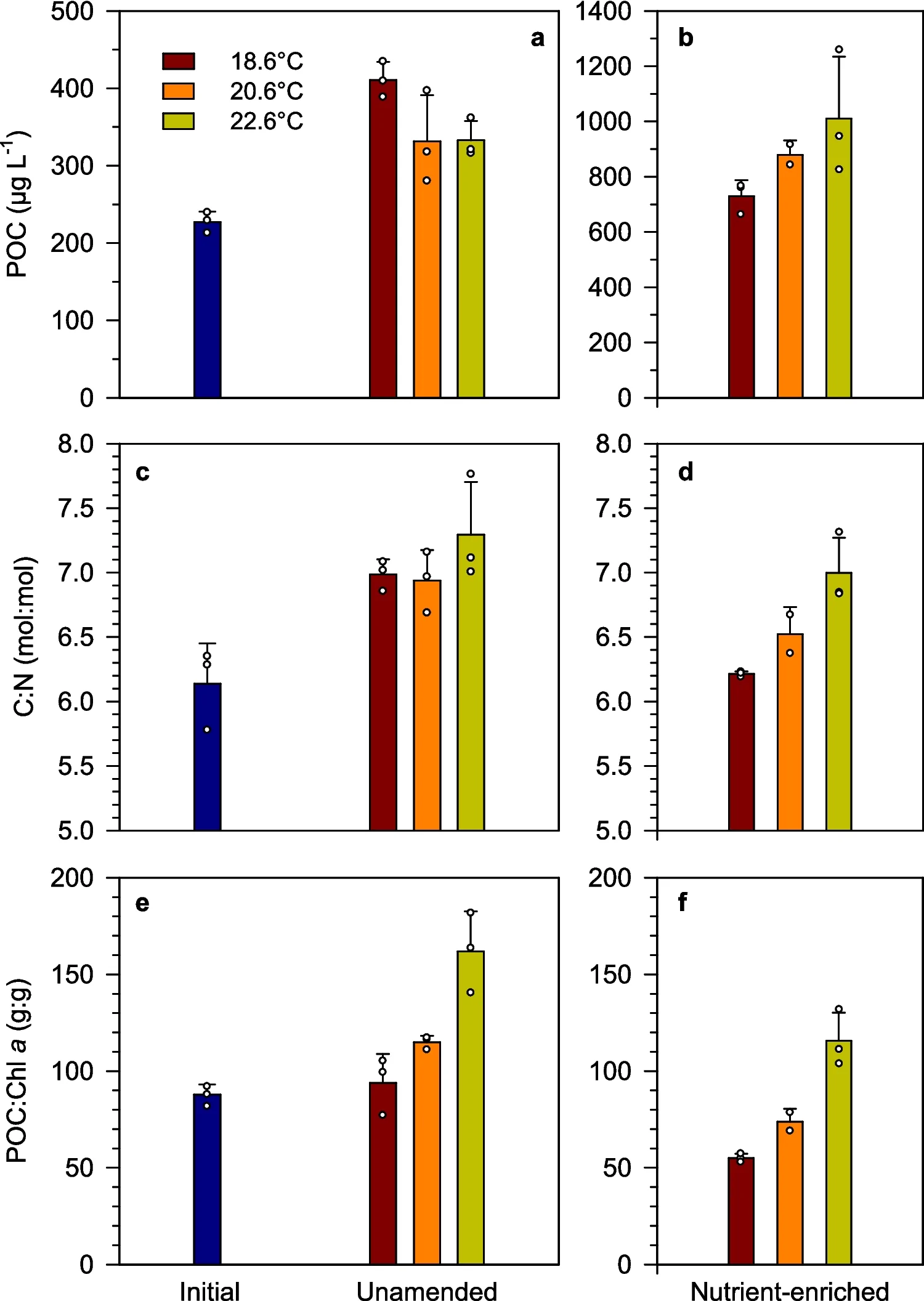
Concentration of **a**, **b** particulate organic carbon (POC), **c**, **d** the carbon-to-nitrogen ratio of particulate organic matter (C:N), and **e**, **f** the POC to chlorophyll *a* ratio (POC:Chl *a*) at the start (initial) and on the last day of the experiment in the different treatments. Note the different Y-axis scale in the POC plots. Small dots represent the values of each biological replicate

The concentration of particulate organic carbon (POC) increased from an initial value of 225 to 300–400 µg L^−1^ in unamended treatments and 700–1000 µg L^−1^ in the nutrient-enriched units (Fig. 2a, b). There was a strong covariation between POC and particulate organic nitrogen (PON), such that PON [µg L^−1^] = 0.17 × POC [µg L^−1^] − 0.61 (*r*^2^ = 0.98, *n* = 20, *p* < 0.001). Compared to the in situ temperature, the warmest treatments tended to show lower POC in the low-nutrient regime and higher POC under nutrient-enriched conditions. Thus, the highest POC value (1000 µg L^−1^) was measured in the nutrient-enriched treatment at 22.6 °C (Fig. 2b). Despite the strong covariation between POC and PON, the molar C:N of particulate organic matter (which took an overall mean value of 6.7 ± 0.5) did show some differences among treatments and over time (Fig. 2c, d). Under low-nutrient conditions, C:N increased at all temperatures from an initial value of 6.1 to values ≥ 7.0 (Fig. 2c). C:N was similar in all temperature treatments under low nutrient conditions but increased with temperature in the nutrient-amended treatments (Fig. 2c, d). At all temperatures, nutrient-enriched treatments showed lower C:N values than their low-nutrient counterparts. The POC:Chl *a* ratio, which depends both on the volume-specific cellular chl *a* content and the contribution of photoautotroph biomass to total particulate organic matter, also displayed differences among treatments at the end of the experiment (Fig. 2e, f). Irrespective of temperature, POC:Chla was always higher in low-nutrient treatments than in nutrient-enriched ones, while it increased with warming under both nutrient regimes (K-W test, *χ*^2^ = 7.2, *p* = 0.027 for unamended treatments and *χ*^2^ = 6.2, *p* = 0.044 for nutrient-enriched ones).

### Picoplankton Biomass

Heterotrophic bacteria and photoautotrophic picoplankton (*Synechococcus* plus the picoeukaryotes) each had similar initial biomass (ca. 12 µg C L^−1^), but over the course of the experiment, the latter increased much more markedly (Table S2). This was mainly due to the small and large picoeukaryotes, which on day 5 reached a biomass of 17–27 µg C L^−1^ and 80–104 µg C L^−1^, respectively, in the nutrient-amended treatments. Small and large picoeukaryotes increased their biomass by a factor of 25 and 10, respectively, while the biomass increase of *Synechococcus* and heterotrophic bacteria was more modest (< threefold). The biomass of both small and large picoeukaryotes was higher in the nutrient-amended treatments at all temperatures, while the biomass of heterotrophic bacteria and *Synechococcus* showed smaller differences between nutrient regimes. Consistent with the pattern described above for chl *a*, warming accelerated the net growth of the large picoeukaryotes under nutrient-enriched conditions, so that on day 3, their biomass at 22.6 °C (70 µg C L^−1^) was significantly higher than at 18.6 °C (30 µg C L^−1^) (K-W test, *χ*^2^ = 7.2, *p* = 0.027; Dunn’s test, *Z* =  − 2.7, *p* = 0.011). Warming also affected the changes in picoeukaryote biomass from day 3 to day 5 in both nutrient regimes, resulting in a decrease or a smaller increase than observed at in situ temperature. This effect was particularly noticeable in the nutrient-enriched treatments, where from day 3 to day 5, large picoeukaryote biomass increased by 2–threefold at 18.6 °C and 20.6 °C but only by 14% at 22.6 °C (Table S2).

### Photochemical Efficiency of Photosystem II

**Fig. 3:**
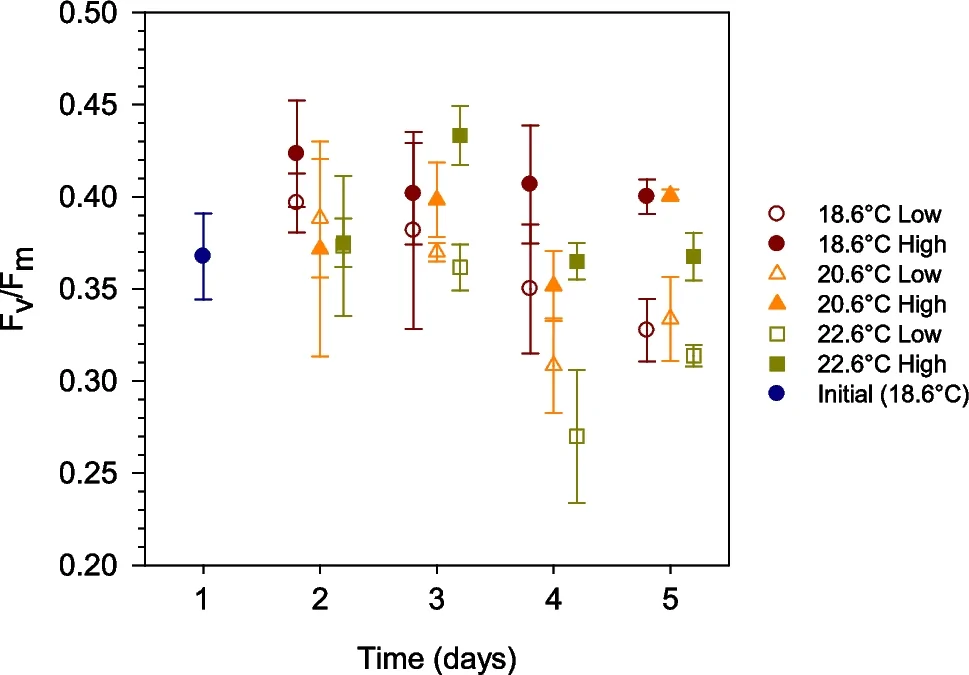
Temporal variability of the maximum photochemical efficiency of photosystem II (*F*_v_/*F*_m_) in the different treatments. Open and closed symbols correspond to unamended (low) and nutrient-enriched (high) conditions, respectively

The maximum potential quantum efficiency of photosystem II (*F*_v_/*F*_m_) was 0.37 ± 0.02 and did not show clear differences between treatments on day 1 (Fig. 3). However, from day 3 onwards and particularly on days 4 and 5, *F*_v_/*F*_m_ was significantly higher in nutrient-enriched than in unamended treatments at all temperatures (ART test, *F*_*N*_ = 37.0, *p* < 0.0001). The effect of temperature was less marked, but on days 4 and 5, *F*_v_/*F*_m_ at 22.6 °C was lower than at 18.6 °C under both nutrient regimes.

### Carbon Fixation and Nitrate Uptake

**Fig. 4:**
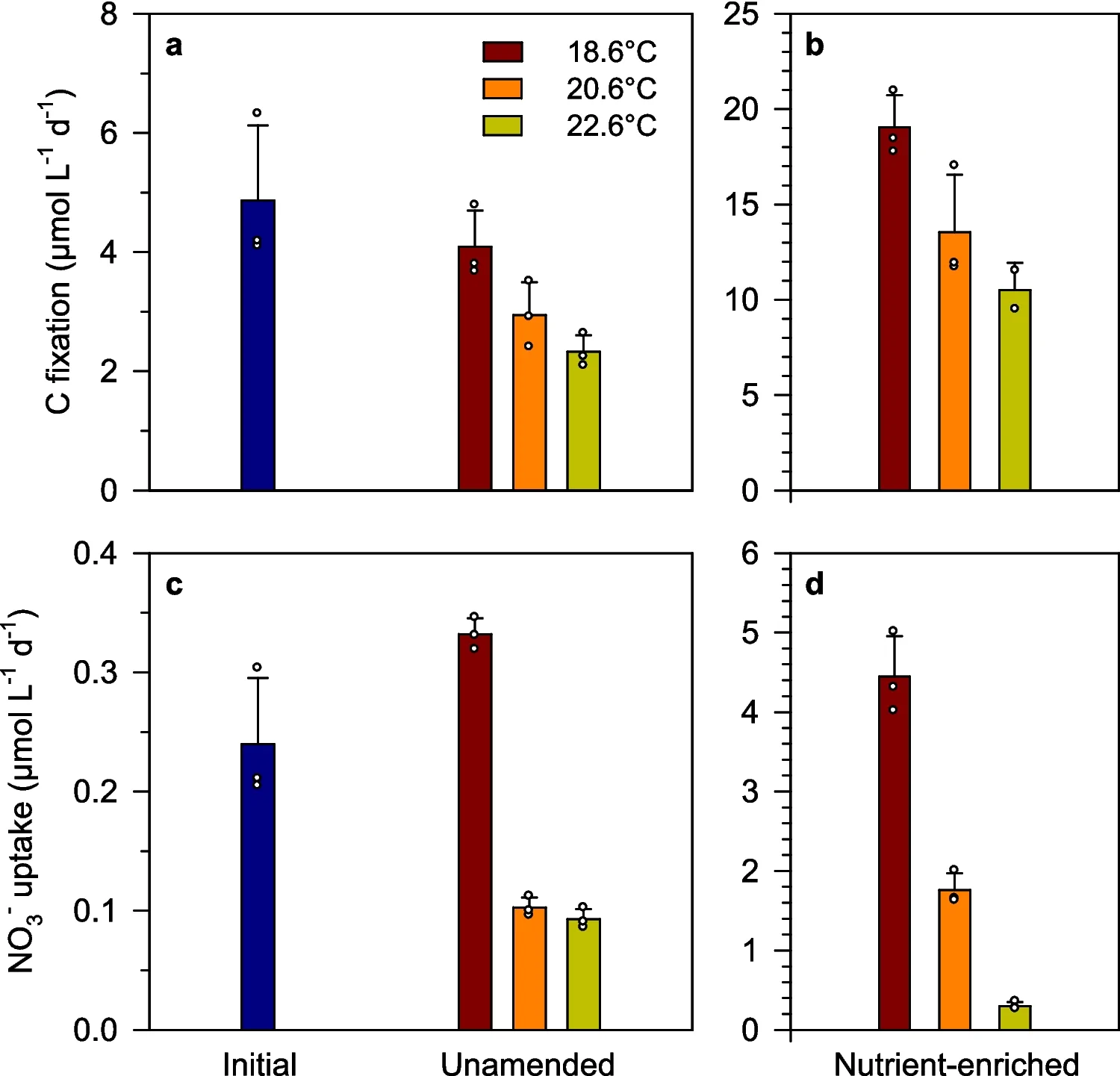
Rates of **a**, **b** photosynthetic CO_2_ fixation and **c**, **d** NO_3_^−^ uptake rates at the start (initial, 18.6 °C) and on the last day of the experiment in the different treatments. Note the differences in the y-axis scale between the panels on the left (initial and unamended treatments) and the panels on the right (treatments with added nutrients). Small dots represent the values of each biological replicate

The rates of photosynthetic carbon fixation and nitrate uptake were much higher (by a factor of 5–10) under nutrient-rich conditions than in the unamended treatments (Fig. 4). Within each nutrient regime, both rates took the highest and lowest values at the in situ and warmest temperatures, respectively. The differences between temperatures and between nutrient treatments were all statistically significant (ART test, *p* ≤ 0.0005). The effect of temperature on nitrate uptake rates was stronger in the nutrient-enriched treatments than in the unamended units (Fig. 4c, d). Thus, we found strong evidence for a temperature × nutrient interactive effect on nitrate uptake rates (ART test, *F*_*T* × *N*_ = 114.2, *df* = 2, *p* < 0.0001). The overall variability among treatments was higher for nitrate uptake than for carbon fixation. As a result, the carbon fixation to nitrate uptake ratio (*C*_fix_:*N*_upt_) ranged widely between 4 and 39 µmolC µmolN^−1^ (Table S3). At 18.6 °C and 20.6 °C, *C*_fix_:*N*_upt_ was higher in the unamended bottles than in those that had been supplemented with nutrients. Under both unamended and nutrient-enriched conditions, the lowest values of *C*_fix_:*N*_upt_ were measured at 18.6 °C, whereas the highest value (38.9 µmolCµmolN^−1^) occurred in the enriched treatment at 22.6 °C.

### Metabolic Balance

**Fig. 5:**
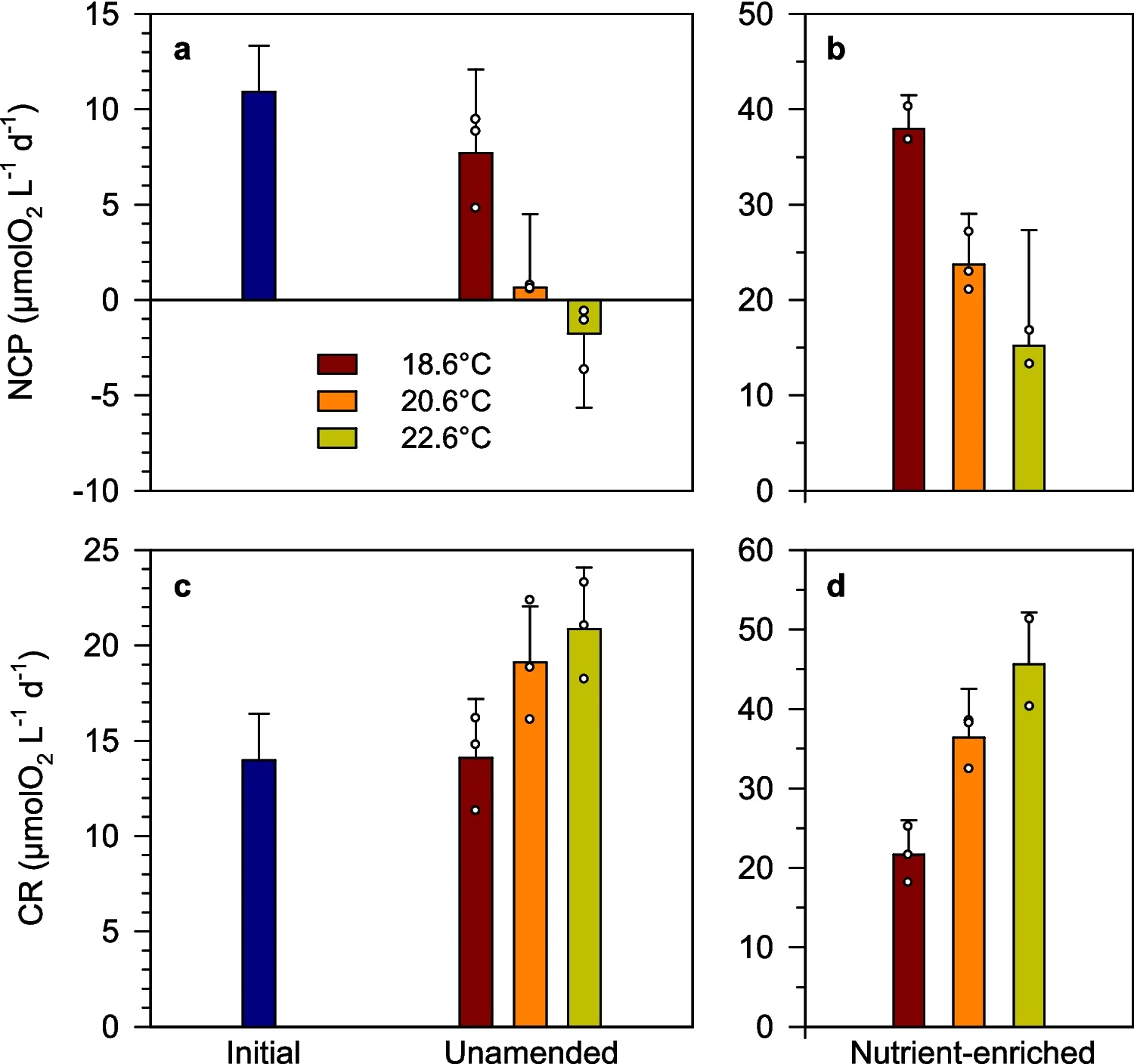
Rates of **a**, **b** net community production (NCP) and **c**, **d** community respiration (CR) at the start (initial, 18.6 °C) and on the last day of the experiment in the different treatments. Note the differences in the y-axis scale between the panels on the left (initial and unamended treatments) and the panels on the right (treatments with added nutrients). Small dots represent the values of each biological replicate

Both net community production (NCP) and community respiration (CR) were higher in the nutrient-enriched treatments than in unamended ones at all temperatures (Fig. 5). Increasing temperature, which gave way to accelerated bloom development and decay in both nutrient regimes, resulted in progressively lower NCP and higher CR, with the metabolic balance even becoming negative (NCP < 0) at 22.6 °C in the unamended treatment (Fig. 5c). There was evidence for a temperature × nutrient interactive effect on CR, which increased from the 18.6 °C to the 22.6 °C treatment by 50% in the unamended units and by > 140% in the nutrient-enriched ones (Fig. 5c, d; ART test, *F*_*T* × *N*_ = 8.5, *p* = 0.005). Gross primary production (GPP) was invariant with respect to temperature (approximately 20 and 60 µmolO_2_ L^−1^ day^−1^ in unamended and nutrient-enriched treatments, respectively) (Table S4).

### Specific Metabolic Rates

**Fig. 6:**
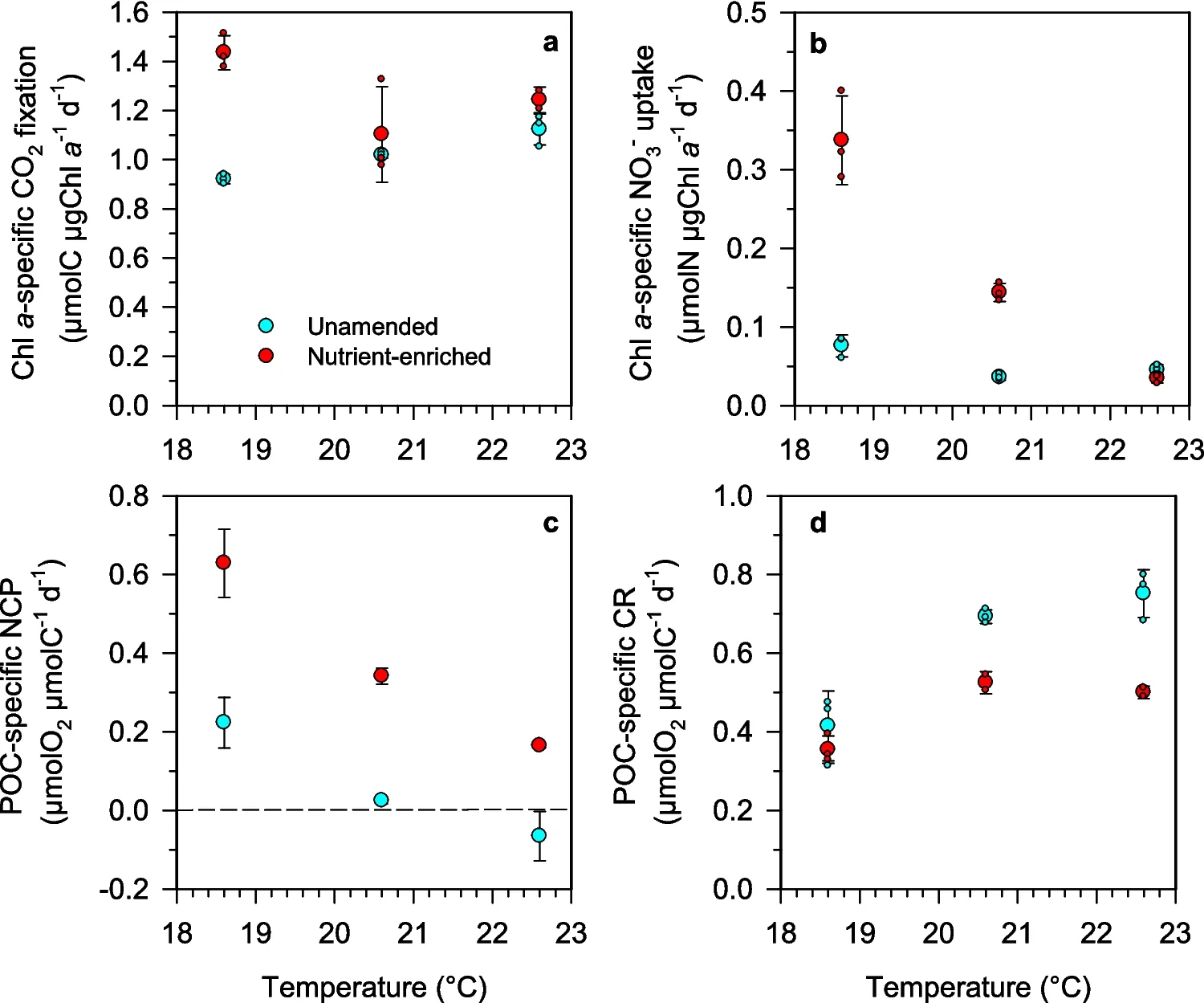
Relationship between temperature and **a** Chl *a*-specific CO_2_ fixation rate, **b** Chl *a*-specific NO_3_^−^ uptake, **c** particulate organic carbon-specific net community production (NCP), and **d** particulate organic carbon-specific community respiration (CR) for unamended (blue circles) and nutrient-amended (red circles) treatments. Small circles indicate the individual data points from each biological replicate. Small dots represent the values of each biological replicate

To examine the impact of the experimental treatments on metabolic rates after removing the effect of changes in photoautotroph abundance and biomass, we calculated chl *a*-specific carbon fixation and nitrate uptake rates (Fig. 6a, b). It must be noted that, because the chl *a* content of cells is itself dependent on temperature and nutrient availability, chl *a*-specific rates must be interpreted with caution (see the “Discussion” section). Considering that both auto- and heterotrophic organisms drive net community production and community respiration, we used POC-specific NCP and CR (Fig. 6c, d) to assess the effect of the experimental treatments on these metabolic rates after removing the influence of changes in microbial biomass. The rates of chl *a*-specific carbon fixation and nitrate uptake and POC-specific NCP all were higher in the nutrient-enriched treatments than in unamended ones (Fig. 6a, b, c). The differences were particularly marked for nitrate uptake (ART test, *F*_*N*_ = 39.5, *p* < 0.0001) and NCP (ART test, *F*_*N*_ = 33.1, *p* < 0.0001). Conversely, POC-specific CR was higher in the unamended treatments (Fig. 6d; ART test, *F*_*N*_ = 29.5, *p* = 0.0003). POC-specific GPP was higher in the nutrient-enriched treatments than in unamended ones at 18.6 °C and 20.6 °C but not at 22.6 °C, where nitrate levels were ≤ 0.1 µmol L^−1^ in both nutrient regimes. The effect of temperature was highly variable depending on the metabolic rate considered. Increasing temperature was associated with a significant decrease in specific nitrate uptake and NCP (Fig. 6b, c; K-W tests; *p* < 0.05), which were lower values at 22.6 °C than at 18.6 °C in both nutrient regimes (Dunn’s tests, *p* < 0.05). On the other hand, there was comparatively little temperature dependence of chl *a*-specific carbon fixation (Fig. 6a). We found evidence for a significant temperature × nutrient interactive effect on chl *a*-specific nitrate uptake, which showed a much stronger decrease with increasing temperature in the nutrient-amended treatments than in the unamended ones (Fig. 6b; ART test, *F*_*T* × *N*_ = 57.5, *p* < 0.0001). POC-specific CR was enhanced by warming in both nutrient regimes, as higher values were measured at 20.6 °C and 22.6 °C than at 18.6 °C (Fig. 6d). However, similar rates of POC-specific CR were found at 20.6 and 22.6 °C, suggesting a saturating effect of warming on community respiration. POC-specific GPP was relatively invariant with temperature in the unamended treatments but showed a decreasing trend with warming in the nutrient-enriched treatments (Table S4).

## Discussion

### Context for the Experiment

Compared to the Ría de Vigo climatology, the environmental setting for our experiment was characterized by warm and low-nutrient conditions. The observed surface temperature, nitrate concentration, and chl *a* concentration contrasted markedly with the climatological values for mid-September (18.6 vs 16.5 °C, 0.5 vs 2 µmol L^−1^, and 2.5 vs 8 µg L^−1^, respectively) [54, 63]. The 2 °C warming treatment corresponded to a temperature of 20.6 °C, which already exceeds the highest recorded surface temperature of the central part of the Ría de Vigo during the last two decades (20 °C) [63, 64], while the 4 °C warming resulted in a temperature of 22.6 °C, which is > 2.5 °C higher than the annual peak. Organisms adapted to warm-to-cool transition periods are more vulnerable to heatwaves than those adapted to seasons with warming trends [65]. In the specific case of phytoplankton, populations exposed to warm temperatures undergo nutrient depletion, which slows down growth and restricts performance during subsequent exposure to high temperatures [66]. There is also experimental evidence that nutrient limitation causes a decrease in *T*_opt_, the optimal temperature for growth, so that populations living in nutrient-impoverished conditions are expected to be more vulnerable to episodes of warming [67]. All of the above would seem to set the stage for a negative impact of experimental warming on phytoplankton, with a decrease in nutrient consumption, carbon fixation, and biomass production. However, the opposite occurred.

### Impacts on Bloom Magnitude and Timing

Warming resulted in an increase in nutrient consumption and biomass production rates (as reflected in the net accumulation of chl *a* and POC over the course of the experiment), and therefore accelerated bloom dynamics both in unamended and nutrient-enriched treatments, although the stimulation was larger in the latter. The observation that the stimulation of phytoplankton net growth by warming was stronger under high-nutrient conditions is consistent with previous laboratory and field experiments [35–37, 39]. The largest concentration (1000 µg C L^−1^) of particulate organic carbon (POC), measured in nutrient-amended microcosms at the warmest temperature (22.6 °C), implied a net rate of increase of 0.37 day^−1^ over a 4-day period, and was paralleled by a nitrate net consumption of approximately 6 µmol L^−1^ in 48 h (from day 3 to day 5). In contrast with earlier studies with natural communities, the warmest temperature used in our experiments exceeded by > 2.5 °C the annual peak recorded in situ. Therefore, the fast and positive responses observed, both in terms of nutrient consumption and net biomass production, indicate that the community was remarkably robust to the thermal perturbation. Although initial nutrient concentrations were relatively low, remineralization of organic matter is intense in the Ría de Vigo, particularly during periods of upwelling relaxation in summer [68, 69]. This provides a continuous source of recycled nutrients that may alleviate the risk of depletion of intracellular pools, thus helping phytoplankton to cope with anomalously warm conditions.

Similar to other experiments with coastal plankton assemblages [70, 71], we found that the development and subsequent decay of the phytoplankton bloom was accelerated by warming. The chl *a* increase observed from day 1 to day 3 in nutrient-enriched units was higher at 22.6 °C than at 18.6 °C by a factor of three. The differences in bloom timing can be appreciated by considering the changes in the particulate organic carbon to chl *a* ratio (POC:Chl *a*), taking also into account the temperature and nutrient dependence of phytoplankton chl *a* content [72, 73]. Cells tend to become richer in chl *a* with increasing temperature and increasing nitrogen availability. However, a 4 °C increase in temperature causes only a relatively modest increase in chl *a* content [39, 73], and, given that in our experiments warming led to faster consumption and lower final concentrations of nitrate, the two effects may have counterbalanced each other to some extent. This would make chl *a* concentration a reasonably good proxy for phytoplankton carbon, and the POC:Chl *a* ratio a valid indicator of the contribution of phytoplankton to total particulate organic matter. Nutrient addition gave way to a marked increase in the photoautotrophic share of organic matter, because at all temperatures POC:Chl *a* decreased by approximately 40% in the amended treatments relative to the unamended ones. The POC:Chl *a* value measured in the nutrient-enriched treatments at 18.6 °C on day 5 (55 µg C µg Chl *a*^−1^) was only slightly higher than the phytoplankton carbon to chl *a* ratio measured in September in coastal waters of the North Atlantic (45–50 µg C µg Chl *a*^−1^) [74, 75]. This suggests that, at the end of the experiment, phytoplankton contributed most of the organic matter in the nutrient-enriched treatments maintained at in situ temperature. In the 20.6 °C and 22.6 °C treatments, however, the bloom was already in a state of decline, which resulted in progressively higher POC:Chl *a* ratios, signaling an increase in the contribution of non-phytoplankton material to total suspended organic carbon.

The observation that warming resulted in faster nutrient consumption and phytoplankton increase, together with the higher final biomass attained in the warmest treatments, does not support the hypothesis that increased temperatures lead to stronger grazing pressure by microzooplankton, which has been put forward as an explanation for the greater frequency of algal blooms in high latitudes [53]. It may be that the relatively strong temperature dependence of microzooplankton growth (relative to that of phytoplankton growth) measured under optimal conditions in the laboratory is not attained in the field, due to food limitation and top-down control among other factors [76]. In addition, when thermal sensitivities are analysed in detail and in the context of changes in phytoplankton community composition, the differences between photoautotrophs and heterotrophic protists appear to be small [77]. As noted by Sherr et al. [76], microzooplankton are unlikely to prevent the onset and development of phytoplankton blooms, provided that resource availability is sufficient to sustain fast photoautotrophic growth. On the other hand, some studies have suggested that experimental warming can lead to increased phytoplankton-zooplankton trophic coupling and decreased algal biomass under nutrient-rich conditions [34, 78]. The short duration of our experiment may have prevented these trophic mechanisms from fully playing out. Grazing by protists is known to be a major loss term for phytoplankton in productive coastal ecosystems such as the Ría de Vigo [79], and additional observations and experiments throughout the year are required to determine the impacts of temperature on the coupling between photoautotrophs and their microbial consumers [80].

### Changes in Carbon Fixation, Nitrate Uptake, and C:N Ratio

Both the volumetric carbon fixation rates and the estimates of photosystem II maximum quantum yield (*F*_v_/*F*_m_) suggest that nutrient supply was a stronger determinant of photosynthesis than temperature. Chl *a*-specific C fixation was always higher in the nutrient-enriched treatments (particularly at 18.6 °C, which had the highest nitrate and silicate concentrations), but these differences must have been larger in terms of carbon turnover rates because phytoplankton C:Chl *a* ratio at a given temperature is lower under increased nitrogen availability [72]. Temperature did impact photosynthetic activity through enhanced nutrient depletion under warming, which resulted in a decrease in carbon fixation under both nutrient regimes. In comparison with carbon fixation, nitrate uptake showed much larger variability among the various treatments, both in terms of volumetric and specific rates. The large contrast in nitrate uptake rate at in situ temperature between unamended and nutrient-enriched microcosms suggests that the differences observed between temperatures in the latter were mostly a result of changes in nitrate availability, itself a result of thermal-induced changes in the pace of nutrient consumption and bloom evolution. Because of the confounding effect of changes in nitrate concentration, an accurate examination of the temperature dependence of nitrate uptake rates would have required kinetic uptake experiments [81]. However, the fact that similar chl *a*-specific rates were measured in all the unamended treatments suggests that nitrate uptake was primarily controlled by nitrate availability rather than by temperature.

Consistent with previous studies that have concurrently investigated CO_2_ fixation and nitrate uptake [82–84], we found a highly variable (> tenfold) carbon fixation to nitrate uptake ratio. This result illustrates the ability of phytoplankton assemblages to take up nutrients in excess of requirements [85, 86] and, conversely, to sustain photosynthetic carbon fixation even after nutrients have been nearly exhausted [87, 88], thus demonstrating the importance of incorporating phytoplankton stoichiometric plasticity into marine biogeochemical models [89, 90]. The C:N of particulate organic matter reflected, to some extent, the dynamics of carbon fixation and nitrate uptake. For instance, the lowest C:N values (6.2–6.5) were measured at 18.6 °C and 20.6 °C in nutrient-enriched treatments, coinciding with the lowest C fixation to nitrate uptake ratios. C:N showed few differences among the various incubation temperatures in unamended microcosms while being consistently lower under nutrient-enriched conditions at all temperatures. This suggests that nutrient availability overrides temperature as a controlling factor of seston C:N, as has been found before in observational [91] and modelling studies [49]. The increasing trend of C:N with warming observed in the nutrient-amended treatments can be attributed to growing nitrogen limitation as the bloom decline progressed, together with faster remineralization of nitrogen over carbon [92, 93]. In those regions where sea surface warming leads to decreased nitrogen supply to the euphotic layer, the resulting increase in organic matter C:N may enhance the carbon export efficiency of the biological pump, which represents a negative feedback of ocean biology on climate change [12].

### Temperature-Nutrient Effects on Metabolic Balance

Warming induced a shift towards a negative or less positive metabolic balance, both as a result of decreased photosynthesis and increased community respiration (CR). This result agrees with previous observations during marine heatwaves [25, 94] as well as with the findings of temperature manipulation experiments [70, 95, 96]. In contrast to carbon fixation and nitrate uptake, CR was consistently stimulated by increasing temperature under both nutrient regimes, supporting the prediction that heterotrophic processes have a stronger thermal sensitivity than autotrophic ones [51, 52]. Specific respiration rates were higher in the unamended treatments, probably because of a larger contribution of non-photosynthetic biomass, as suggested by the higher POC:Chl *a* ratios. However, the thermal dependence of POC-specific respiration was similar under both nutrient regimes, which is consistent with the results of [97], who found that the relationship between temperature and bacterial respiration (a major component of total microbial respiration) has the same slope under different levels of resource availability. The positive relationship between temperature and CR is often used to argue that a warming ocean will result in a decreased (or even negative) metabolic balance [52, 98]. However, respiration is also constrained by the supply of resources, and ultimately ecosystem respiration depends closely on primary productivity [99]. In our experiment, we found that the relationship between temperature and POC-specific CR was non-linear, with similar rates measured at 20.6 °C and 22.6 °C, likely reflecting a decreasing availability of fresh, labile organic substrates as the phytoplankton bloom declines.

The observed differences in metabolic balance between temperatures must be interpreted in the context of the different timing of phytoplankton bloom onset and decline in each set of microcosms. Warming resulted in decreased net community production at the end of the experiment under nutrient-enriched conditions, but the overall net POC accumulation and nutrient consumption over the 5-day duration of the experiment were positively related to temperature. Although additional experiments conducted in other seasons are required to determine if these responses are recurrent throughout the year, our results suggest that short-term warming events have the potential to increase nutrient removal, biomass production, and CO_2_ drawdown by microbial plankton in this temperate and productive ecosystem. This conclusion, however, refers only to the biological response of the planktonic community to the warming conditions. Our findings also highlight the preeminent role of nutrient availability in controlling key microbial fluxes such as carbon fixation, nitrogen uptake, and net oxygen production. To the extent that marine heatwaves in coastal embayments are associated with elevated seawater temperatures in the continental shelf [100] and given the general inverse correlation between temperature and nutrient concentration in the surface ocean, these warm events may be associated with reduced nutrient supply, which could depress planktonic productivity. Determining the net impact of marine heatwaves on the plankton of the Rías Baixas and other coastal ecosystems will require in situ studies that address both the coast-shelf nutrient exchange and the ecophysiological response of the community.

## Supplementary Information

Below is the link to the electronic supplementary material.

ESM 1 (DOCX 1.63 MB)

## Data Availability

**The datasets generated during the current study are available from the corresponding author on reasonable request.**
