# Supplementary material for: Warming Accelerates Phytoplankton Bloom Dynamics and Differentially Affects the Fluxes of Carbon, Nitrogen, and Oxygen Through a Coastal Microbial Community
Source: Microb Ecol. 2025 Nov 7;88(1):117. doi: 10.1007/s00248-025-02643-9 (PMC12594743; doi:10.1007/s00248-025-02643-9)
Supplement: Supplementary file 1 — (DOCX 1.63 MB) [file 248_2025_2643_MOESM1_ESM.docx]

**Supplementary material**

**Warming accelerates phytoplankton bloom dynamics and differentially affects the fluxes of carbon, nitrogen and oxygen through a coastal microbial community**

Daffne C. López-Sandoval, Cristina Fernández-González, Cristina González-García, Emilio Marañón

www.indicedeafloramiento.ieo.es


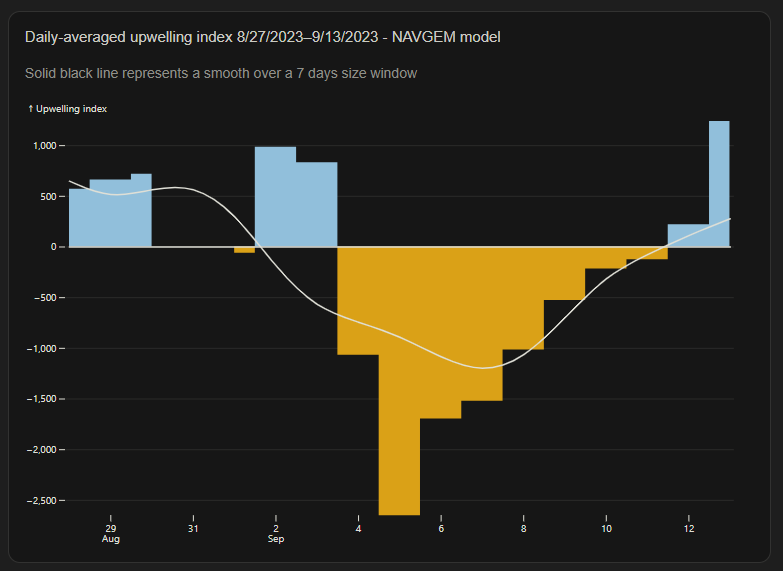


**Figure S1.** Daily-averaged upwelling index (*UI*) during the period Aug 28th – Sep 13th, 2023. Positive (blue) and negative (yellow) values of *UI* correspond to upwelling- and downwelling-favourable conditions, respectively. The white line represents the 1-week moving average. The dashed green line marks the sampling day (Sep 11th, 2023). *UI* was computed by Centro Oceanográfico de Vigo (Instituto Español de Oceanografía) as the offshore Ekman transport perpendicular to the coast: $UI=- \frac{\rho\text{a} C\text{d} V V\text{y}}{\rho\text{sw} f}$, where $\rho\text{a}$ and $\rho\text{sw}$ are the density of air and seawater, respectively, $C\text{d}$ is the drag coefficient, $V$ is the wind speed, $V\text{y}$ is the magnitude of the northerly wind component, and $f$ is the Coriolis parameter at 43°.

**Figure S2.** Vertical profiles of temperature, salinity and chl-*a* fluorescence at the central station of Ría de Vigo on Sep 11, 2023.

**Table S1.** Concentration (µmol L^-1^) of nitrate, nitrite, phosphate and silicate in each treatment. Mean and standard deviation (*n* = 3) are given.

| Day | Temp. °C | Nutrient treatment | Nitrate | | Nitrite | | Phosphate | | Silicate | |
| --- | --- | --- | --- | --- | --- | --- | --- | --- | --- | --- |
|  |  |  | Mean | SD | Mean | SD | Mean | SD | Mean | SD |
| 1 | 18.6 | Unamended | 0.51 | 0.17 | 0.15 | 0.02 | 0.29 | 0.04 | 1.17 | 0.32 |
| 1 | 18.6 | Enriched | 6.44 | 0.19 | 0.14 | 0.03 | 0.46 | 0.05 | 4.63 | 0.18 |
| 3 | 18.6 | Unamended | 0.18 | 0.01 | 0.09 | 0.03 | 0.25 | 0.01 | 0.93 | 0.24 |
| 3 | 18.6 | Enriched | 6.21 | 0.12 | 0.13 | 0.01 | 0.26 | 0.01 | 5.38 | 0.15 |
| 3 | 20.6 | Unamended | 0.04 | 0.02 | 0.03 | 0.02 | 0.21 | 0.03 | 0.55 | 0.04 |
| 3 | 20.6 | Enriched | 6.08 | 0.25 | 0.14 | 0.03 | 0.28 | 0.00 | 5.88 | 0.36 |
| 3 | 22.6 | Unamended | 0.03 | 0.02 | 0.03 | 0.01 | 0.23 | 0.02 | 0.35 | 0.04 |
| 3 | 22.6 | Enriched | 5.83 | 0.07 | 0.13 | 0.02 | 0.24 | 0.00 | 5.01 | 0.21 |
| 5 | 18.6 | Unamended | 0.11 | 0.01 | 0.05 | 0.01 | 0.88 | 0.07 | 0.26 | 0.07 |
| 5 | 18.6 | Enriched | 2.86 | 0.63 | 0.11 | 0.01 | 1.11 | 0.07 | 1.30 | 0.17 |
| 5 | 20.6 | Unamended | 0.03 | 0.00 | 0.10 | 0.02 | 1.11 | 0.25 | 0.31 | 0.05 |
| 5 | 20.6 | Enriched | 0.82 | 0.18 | 0.11 | 0.02 | 0.79 | 0.04 | 0.84 | 0.38 |
| 5 | 22.6 | Unamended | 0.03 | 0.01 | 0.11 | 0.02 | 0.62 | 0.04 | 0.42 | 0.16 |
| 5 | 22.6 | Enriched | 0.10 | 0.02 | 0.04 | 0.02 | 0.39 | 0.09 | 0.59 | 0.25 |

**Table S2.** Biomass (µgC L^-1^) of heterotrophic bacteria (HB), *Synechococcus* (*Syn*), small picoeukaryotes (s_Pico) and large picoeukaryotes (l_Pico) in each treatment. Mean and standard deviation (*n* = 3) are given.

| Day | Temp. °C | Nutrientes treatment | HB | | *Syn* | | s_Pico | | l_Pico | |
| --- | --- | --- | --- | --- | --- | --- | --- | --- | --- | --- |
|  |  |  | Mean | SD | Mean | SD | Mean | SD | Mean | SD |
| 1 | 18.6 | Initial | 12.1 | 1.9 | 0.8 | 0.0 | 1.1 | 0.3 | 10.3 | 2.5 |
| 3 | 18.6 | Unamended | 35.1 | 2.1 | 1.1 | 0.1 | 2.1 | 0.6 | 28.1 | 10.2 |
| 3 | 18.6 | Enriched | 37.7 | 3.8 | 1.2 | 0.0 | 2.6 | 1.2 | 30.6 | 5.2 |
| 3 | 20.6 | Unamended | 26.8 | 0.5 | 1.4 | 0.1 | 2.1 | 0.5 | 35.5 | 8.2 |
| 3 | 20.6 | Enriched | 32.4 | 3.9 | 1.4 | 0.1 | 3.1 | 1.1 | 42.4 | 7.9 |
| 3 | 22.6 | Unamended | 20.0 | 1.7 | 1.2 | 0.0 | 2.3 | 0.5 | 43.1 | 11.1 |
| 3 | 22.6 | Enriched | 27.2 | 1.4 | 1.3 | 0.1 | 3.3 | 2.9 | 70.2 | 6.8 |
| 5 | 18.6 | Unamended | 12.1 | 1.1 | 1.5 | 0.1 | 3.0 | 0.8 | 39.1 | 3.0 |
| 5 | 18.6 | Enriched | 18.1 | 2.9 | 1.6 | 0.2 | 17.5 | 3.5 | 87.3 | 15.0 |
| 5 | 20.6 | Unamended | 16.2 | 0.3 | 2.4 | 0.1 | 1.9 | 0.3 | 26.8 | 7.1 |
| 5 | 20.6 | Enriched | 19.7 | 1.7 | 2.5 | 0.2 | 27.3 | 4.5 | 104.4 | 7.2 |
| 5 | 22.6 | Unamended | 17.7 |  | 1.2 | 0.3 | 1.5 | 0.5 | 22.2 | 6.1 |
| 5 | 22.6 | Enriched | 25.8 | 14.5 | 1.9 | 0.0 | 26.0 | 5.1 | 80.0 | 14.7 |

**Table S3.** Photosynthetic carbon fixation to NO_3_^-^ uptake ratio (µmolC µmolN^-1^) in each treatment. Mean and standard deviation (*n* = 3) are given.

| Day | Temperature °C | Nutrient treatment | CO_2_ fixation to NO_3_^-^ uptake ratio | |
| --- | --- | --- | --- | --- |
|  |  |  | Mean | SD |
| 1 | 18.6 | Unamended | 20.2 | 0.7 |
| 5 | 18.6 | Unamended | 12.4 | 2.3 |
| 5 | 18.6 | Enriched | 4.3 | 0.8 |
| 5 | 20.6 | Unamended | 28.5 | 3.2 |
| 5 | 20.6 | Enriched | 7.6 | 0.8 |
| 5 | 22.6 | Unamended | 25.3 | 5.1 |
| 5 | 22.6 | Enriched | 38.9 | 6.0 |

**Table S4.** Gross primary production (GPP, µmolO_2_ L^-1^ d^-1^) and POC-specific GPP (µmolO_2_ µmolC^-1^ d^-1^) in each treatment. Mean and standard deviation (*n* = 3) are given.

| Day | Temperature °C | Nutrient treatment | GPP | | POC-specific GPP | |
| --- | --- | --- | --- | --- | --- | --- |
|  |  |  | Mean | SD | Mean | SD |
| 1 | 18.6 | Unamended | 24.9 | 3.6 | 1.31 | 0.16 |
| 5 | 18.6 | Unamended | 21.8 | 2.9 | 0.64 | 0.08 |
| 5 | 18.6 | Enriched | 59.6 | 2.1 | 0.98 | 0.07 |
| 5 | 20.6 | Unamended | 19.8 | 3.0 | 0.72 | 0.02 |
| 5 | 20.6 | Enriched | 60.1 | 6.2 | 0.87 | 0.01 |
| 5 | 22.6 | Unamended | 19.1 | 4.1 | 0.69 | 0.12 |
| 5 | 22.6 | Enriched | 60.8 | 10.3 | 0.66 | 0.02 |
